# Chemotherapy *vs* supportive care alone for relapsed gastric, gastroesophageal junction, and oesophageal adenocarcinoma: a meta-analysis of patient-level data

**DOI:** 10.1038/bjc.2015.452

**Published:** 2016-02-16

**Authors:** Tobias Janowitz, Peter Thuss-Patience, Andrea Marshall, Jung Hun Kang, Claire Connell, Natalie Cook, Janet Dunn, Se Hoon Park, Hugo Ford

**Affiliations:** 1 Department of Oncology, Addenbrooke's Hospital, Box 193, Hills Road, Cambridge CB2 0QQ, UK; 2 Charité–Universitätsmedizin Berlin, Medizinische Klinik mit Schwerpunkt Hämatologie, Onkologie und Tumorimmunologie, Augustenburger Platz 1, 13353 Berlin, Germany; 3 Warwick Medical School Clinical Trials Unit, University of Warwick, Coventry CV4 7AL, UK; 4 Department of Internal Medicine, School of Medicine, Gyeongsang National University, Jinju 52727, Republic of Korea; 5 Division of Hematology-Oncology, Department of Medicine, Sungkyunkwan University Samsung Medical Center, Seoul 135-710, South Korea

**Keywords:** gastric cancer, second-line chemotherapy, supportive care, meta-analysis, patient-level data, age

## Abstract

**Background:**

Second-line chemotherapy treatment of patients with relapsed gastric and oesophageal cancers in comparison with supportive care (SC) alone has been supported by recent phase 3 clinical trials, but a meta-analysis of patient-level data is lacking.

**Methods:**

We searched Medline, the Cochrane Central Register of Controlled Trials (CENTRAL), and the Web of Science for phase 3 clinical trials that compared second-line chemotherapy with SC alone for gastric and oesophageal cancers. A meta-analysis of the comprehensive patient-level data from the three identified trials was performed.

**Results:**

A total of 410 patients with gastric (*n*=301), gastroesophageal junction (*n*=76), or oesophageal (*n*=33) adenocarcinoma were identified. In all, 154 patients received single-agent docetaxel and 84 patients received single-agent irinotecan, each with SC. SC alone was given to 172 patients. Chemotherapy significantly reduced the risk of death (hazard ratio (HR)=0.63, 95% confidence interval (CI)=0.51–0.77, *P*<0.0001). This effect was observed for treatment with docetaxel (HR=0.71, 95% CI=0.56–0.89, *P*=0.003) and irinotecan (HR=0.49, 95% CI=0.36–0.67, *P*<0.001). Overall survival (OS) benefit was greatest for patients who progressed 3–6 months following first-line chemotherapy (HR=0.39, 95% CI=0.26–0.59, *P*<0.0001). Performance status (PS) 0–1 compared with PS 2 (HR=0.66, 95% CI=0.46–0.94, *P*=0.02), locally advanced disease compared with metastatic disease (HR=0.41, 95% CI=0.25–0.67, *P*=0.0004) and older age (HR=0.94 per 5 years, 95% CI=0.90–0.99, *P*=0.01) were significant predictors of improved OS. Progression of disease during first-line treatment (HR=1.24, 95% CI=0.96–1.59) or within the first 3 months of completion of first-line treatment (HR=1.42, 95% CI=1.09–1.83) were predictors of an increased risk of death compared with progression between 3 and 6 months (*P*=0.03). Health-related quality of life outcomes were reported in only one of the three trials, precluding meta-analysis of these parameters.

**Conclusions:**

This meta-analysis of patient-level data confirms that second-line chemotherapy treatment results in significantly better OS compared with SC alone in patients with platinum and fluoropyrimidine refractory gastric and oesphageal adenocarcinoma. Health-related quality of life outcomes should be included in future trials in this setting.

An evidence base for second-line treatment of gastric and oesophageal cancers is important for the following reasons. First, the global incidence rates are high with 989 000 gastric cancer and 482 000 oesophageal cancer cases yearly, ranking them as the fourth and seventh most common cancer, respectively.(Ferlay *et al*, 2010) Second, most patients are diagnosed with locally advanced or metastatic disease at which point median overall survival (OS) with first-line chemotherapy is only approximately 7–11 months (Wagner *et al*, 2010). Third, after first-line combination treatment including surgery for early-stage disease, the majority of patients relapse (Hartgrink *et al*, 2009). Consequently, gastric and oesophageal adenocarcinoma claim more than a million lives annually and contribute an estimated 15.1% to global cancer mortality (Ferlay *et al*, 2010).

Three phase 3 randomised controlled clinical trials have demonstrated superior OS with second-line irinotecan or docetaxel chemotherapy compared with active symptom control or best supportive care (SC) alone, hereafter referred to as SC (Thuss-Patience *et al*, 2011; Kang *et al*, 2012; Ford *et al*, 2014). A further phase 3 trial compared paclitaxel and irinotecan chemotherapy without a SC arm and described similar OS for both chemotherapies (Hironaka *et al*, 2013). Four phase 3 trials have reported on targeted treatment in the same clinical setting. Everolimus, a mammalian target of rapamycin inhibitor, did not improve OS compared with placebo in patients with advanced gastric cancer progressing after one to two lines of chemotherapy (Ohtsu *et al*, 2013). Gefitinib, an EGFR inhibitor, provided a 0.4 month benefit in progression-free survival and improvement in a selection of health-related quality of life (HRQoL) outcomes, but no OS benefit compared with placebo in patients with oesophageal cancer progressing after up to three lines of chemotherapy (Dutton *et al*, 2014). In contrast, ramucirumab, an anti-vascular endothelial growth factor receptor type 2 antibody, provided an OS benefit comparable to the benefit achieved with chemotherapy (Fuchs *et al*, 2014). Ramucirumab has also been shown to deliver a combination benefit with paclitaxel chemotherapy when compared with single-agent paclitaxel (Wilke *et al*, 2014).

To corroborate the evidence base for second-line treatment with chemotherapy in gastric and oesophageal adenocarcinoma, we performed a comprehensive meta-analysis of the patient-level data of the three relevant phase 3 trials. A previous study has reported preliminary meta-data on hazard ratios (HRs) from results that were in one case incomplete and only published as conference abstract (Kim *et al*, 2013). A second meta-analysis of second-line treatment for gastric cancer, which included ramucirumab single-agent treatment, was based only on trial-level data (Iacovelli *et al*, 2014). Our study provides several new results based on an in depth analysis of patient-level data as well as the definitive results of the meta-analysis, including the definitive results for OS.

## Materials and methods

### Study criteria, search, and selection

This meta-analysis follows PRISMA guidelines (Moher *et al*, 2009). We included all completed and peer review published phase 3 randomised clinical trials that investigated the effect of second-line chemotherapy in comparison with SC for the treatment of gastric and oesphageal adenocarcinoma. We searched PubMed, the Web of Science, and the Cochrane Central Register of Controlled Trials up to the date of 19 August 2015.

We used the search algorithm: clinical trial, phase 3 AND (randomised OR randomised, controlled trial) AND (gastric OR gastroesophageal OR oesophagogastric OR stomach) AND (cancer OR neoplasm OR carcinoma OR malignant OR malignancy) AND (second-line OR salvage OR supportive care OR active symptom control) AND (chemotherapy OR chemotherapeutic OR antineoplastic agent OR therapy). All identified entries were screened for relevance, eligibility, and duplication.

### Data collection and data items

The chief investigators and trial groups of the three identified trials provided comprehensive patient-level data from the original trial databases. Data were sought for the primary outcome of OS as well as for the patient characteristics of sex, age, and Eastern Cooperative Oncology Group (ECOG) performance status (PS). Data on disease status, site of primary disease, and response to previous chemotherapy were collated and analysed as well as data on treatment, including type of chemotherapy, number of administered cycles, reason for end of treatment, best response to first-line chemotherapy, and administration of further chemotherapy after trial participation.

### Summary measures and statistical analysis

The primary outcome of this meta-analysis was OS, calculated as the time from trial entry until death from any cause or censored at the date last known to be alive. Updated follow-up data were obtained compared with the published data (Thuss-Patience *et al*, 2011; Kang *et al*, 2012; Ford *et al*, 2014) and therefore an analysis of the individual trials was performed using Kaplan–Meier survival curves to obtain the median survival and associated 95% confidence interval (CI) using the log-log transformation. For illustration, a Kaplan–Meier survival curve of all trials has been provided in the Supplementary Figure 1. Because this is based on pooled data from the three trials no HRs have been calculated and interpretation is limited (Tierney *et al*, 2015). Heterogeneity between the studies was assessed using the Cochran's Q statistic (Early Breast Cancer Trialists' collaborative group 1990). A one-stage random effects model was used for the analysis of the data.

The patient, disease, and treatment characteristics were evaluated for their prognostic value of OS using a multivariable one-stage random effects Cox proportional hazards model using backwards regression.

Analyses were performed using the SAS statistical software (version 9.3) and R statistical software (version 3.0.3). Results were reported as HR with 95% CIs. Reported *P*-values were two sided, had not been adjusted for multiple testing, and were considered statistically significant at a value of less than 0.05.

### Risk of bias

Clinical trials that had not published results in peer-reviewed medical journals or were not randomised phase 3 trials were not included in this meta-analysis. There is a risk that such trials would have identified different effects on OS. Most smaller studies, retrospective analysis, case series, and case reports, however, indicate benefit of chemotherapy. Reports on such smaller studies had already resulted in use of second-line chemotherapy in patients with relapsed gastric and oesophageal adenocarcinoma, before the establishment of level 1 evidence of OS benefit by phase 3 clinical trials (Ford and Gounaris, 2015).

## Results

The database searches returned 32 entries in PubMed, 30 reports in the Cochrane Register, and 75 entries in the Web of Science. Twenty-nine were duplicate entries. The majority of these 108 identified publications had to be excluded because they were not a randomised phase 3 trial, did not investigate second-line chemotherapy *vs* SC in gastric and oesophageal cancers, or were not peer reviewed. One phase 3 trial compared second-line chemotherapy with paclitaxel against irinotecan without a SC arm (Hironaka *et al*, 2013) and was, therefore, excluded. Three phase 3 trials compared targeted treatment with everolimus (Ohtsu *et al*, 2013), ramucirumab (Fuchs *et al*, 2014), or gefitinib with SC, but were excluded because they did not involve administration of chemotherapy. The RAINBOW trial did not include a SC arm (Wilke *et al*, 2014) and was consequently not included in this analysis. Final manual assessment of all entries resulted in identification of three phase 3 trials that fulfilled eligibility criteria for this meta-analysis (Thuss-Patience *et al*, 2011; Kang *et al*, 2012; Ford *et al*, 2014).

### Study, patient, disease, and primary treatment characteristics

The study, patient, disease, and primary treatment characteristics for the 410 patients of the three individual trials are summarised in Table 1. The trials had a higher proportion of patients who were male compared with female (*n*=302 *vs n*=108), aged below 70 years compared with above 70 years (*n*=348 *vs n*=62), and who had an ECOG PS of 0 or 1 compared with 2 (*n*=373 *vs n*=37). The trial conducted by Ford *et al* (2014) was the only trial to include locally advanced patients and therefore the majority of the patients across the three trials had metastatic disease rather than locally advanced disease (*n*=389 *vs n*=21). The largest of the three trials, conducted by Kang *et al*, only included patients with gastric cancer and overall more patients had gastric rather than gastroesophageal junction or oesophageal cancers (*n*=301 *vs n*=76 *vs n*=33).

A higher proportion of patients had a complete response (CR) or partial response (PR) to previous chemotherapy within the trial reported by Kang *et al* compared with the others (Table 1). Overall, most patients (*n*=199, 49%) had progressive disease (PD) as their best response to previous chemotherapy, 78 patients (19%) had stable disease, 117 patients (29%) had PR, and 6 patients (1%) had CR. A similar number of patients had progressed either during or within 3 or 3–6 months of previous chemotherapy across the three trials. Most patients did not receive treatment in the form of surgery (*n*=305 *vs n*=105) or radiotherapy (*n*=369 *vs n*=41) before enrolment.

### Second-line chemotherapy and SC treatment

The data relating to trial treatments analysed in this meta-analysis are listed in Table 2. Out of the 238 patients who were allocated to chemotherapy plus SC across the three trials, 154 patients received docetaxel and 84 irinotecan. A total of 866 cycles of chemotherapy were administered with a median of three cycles per patient (range 0–12). Most patients stopped treatment because of PD (*n*=165, 69%), followed by unacceptable toxicity (*n*=24, 10%), and completion of treatment (*n*=19, 8%). No case of CR was observed, but 15 patients (6%) had PR, and 75 patients (32%) had stable disease. PD was the best response to chemotherapy in 116 patients (49%).

Assessment of patients on both arms was scheduled at the same frequency in all included trials (Supplementary Table 1). Both arms allowed management of symptoms with analgesia, anti-emetics, steroids, and palliative radiotherapy. Subsequent chemotherapy was permitted in both treatment arms. After trial completion, 97 (24%) of the entire 410 patients (24%) received further chemotherapy: 65 (27%) of the 238 patients allocated to chemotherapy plus SC, 32 (19%) of the 172 patients on the SC arms (Supplementary Table 2).

### Treatment effect on OS

Each of the three trials had a significant reduction in the HR for death and an increased median survival of approximately 2 months with chemotherapy and SC compared with SC alone (Table 3). A Kaplan–Meier curve of patient survival data across all trials is presented in Supplementary Figure 1.

**Figure 1:**
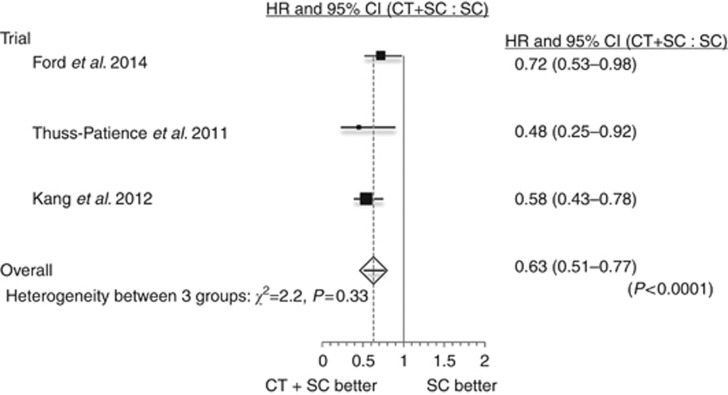
**Forest plot of the hazard ratio (HR) for death of chemotherapy and supportive care (CT+SC) compared with supportive care (SC) alone.** Overall HR from a one-stage random effects Cox regression model.

**Figure 2:**
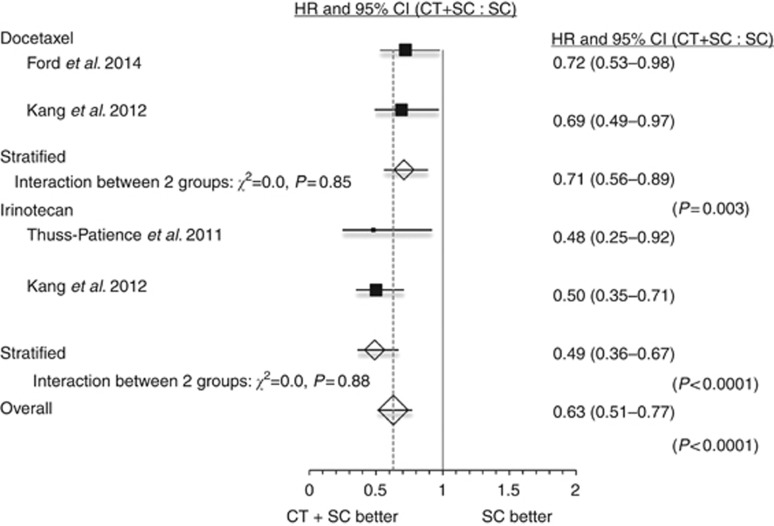
**Forest plot of the hazard ratio (HR) for death with chemotherapy and supportive care (CT+SC) compared with supportive care (SC) alone for trials using docetaxel and those using irinotecan separately.** Overall HR from a one-stage random effects Cox regression model.

Meta-analysis with a one-stage random effects Cox regression model confirmed a highly significant reduction in the risk of death for patients receiving chemotherapy and SC compared with SC alone (HR=0.63, 95% CI=0.51–0.77, *P*<0.0001, Figure 1). This effect was confirmed for chemotherapy use with docetaxel (HR=0.71, 95% CI=0.56–0.89, *P*=0.003) and irinotecan (HR=0.49, 95% CI=0.36–0.67, *P*<0.0001, Figure 2).

### Treatment interactions

The interaction between time to progression (TTP) and treatment is the only significant treatment interaction (*P*=0.04, Table 4), suggesting that the treatment effect was similar across all other covariate subgroups. Patients progressing within 3–6 months after prior chemotherapy tended to have more benefit from second-line chemotherapy (HR=0.39, 95% CI=0.26–0.59, *P*<0.0001) than those who progressed within 3 months of completing treatment (HR=0.70, 95% CI=0.49–0.99, *P*=0.04) or during treatment (HR=0.75, 95% CI=0.54–1.04, *P*=0.08).

### Predictors of OS

In a multivariable one-stage random effects Cox regression model, PS (*P*=0.02), disease stage (*P*=0.0004), TTP after first-line chemotherapy (*P*=0.03), and age (*P*=0.01) were all significant predictors of OS in addition to treatment (*P*<0.0001). Patients with a PS of 0–1 compared with PS 2 (HR=0.66, 95% CI=0.46–0.94), or locally advanced disease compared with metastatic disease (HR=0.41, 95% CI=0.25–0.67) or older age (HR=0.94 per 5 years, 95% CI=0.90–0.99) had an improved OS. In contrast, patients whose disease progressed during first-line treatment (HR=1.24, 95% CI=0.96–1.59) or within the first 3 months of completion of first-line treatment (HR=1.42, 95% CI=1.09–1.83) were more likely to have shorter OS than those who progressed 3–6 months after first-line treatment completion.

## Discussion

Three phase 3 trials have reported a significantly improved OS with second-line chemotherapy and SC compared with SC alone (Thuss-Patience *et al*, 2011; Kang *et al*, 2012; Ford *et al*, 2014). This meta-analysis examined patient-level data from these three trials. It definitively confirms highest level evidence of an OS benefit with second-line chemotherapy (HR=0.63, 95% CI=0.51–0.77, *P*<0.0001). Overall survival benefit is conferred by both docetaxel (HR=0.71, 95% CI=0.56–0.89, *P*=0.003) and irinotecan (HR=0.49, 95% CI=0.36–0.67, *P*<0.0001). These findings consolidate the use of second-line chemotherapy as the standard of care in the management of relapsed gastric and oesphageal adenocarcinoma.

Future clinical trials are required to further improve the outcome for patients with gastric and oesophageal adenocarcinoma. Targeted therapies have shown some promise in recent phase 3 trials. Ramucirumab, a monoclonal antibody targeting vascular endothelial growth factor receptor type 2, increases median OS in relapsed gastric cancer, both as a single-agent relative to placebo in the REGARD trial (Fuchs *et al*, 2014) and in combination with paclitaxel relative to single-agent paclitaxel in the RAINBOW trial (Wilke *et al*, 2014). In contrast, everolimus, for patients progressing after chemotherapy for advanced gastric cancer, did not confer an OS benefit (Ohtsu *et al*). Gefitinib, for patients progressing after chemotherapy for oesophageal cancer, while not conferring an OS benefit, was associated with an improvement in aspects of HRQoL outcomes, including odynophagia, and notable rapid and durable responses in a subpopulation of patients (Dutton *et al*, 2014). Preliminary data from a phase 2 study, communicated in a conference abstract, indicate significant improvement in progression-free survival with regorafenib, a multi-kinase inhibitor, in patients with relapsed gastric and oesophageal cancer (Pavlakis *et al*, 2015). Final data are awaited. In summary, to date, current gains in OS with second-line agents, including combination regimes, remain modest.

Identification of biomarkers or clinical data predictive of patient response to therapy will help select those most likely to benefit from treatment. This meta-analysis has identified TTP after first-line chemotherapy to impact significantly on response to second-line chemotherapy: patients progressing 3–6 months following first-line chemotherapy gained most benefit in OS, whereas there was no significant gain in OS for those patients who progressed during first-line chemotherapy. This finding warrants stratification by TTP from first-line chemotherapy in future studies evaluating second-line chemotherapy and prospective evaluation with other treatment agents and combinations. In contrast, an effect on OS between progression-free interval of less than *vs* more than 6 months was not found with ramucirumab in the REGARD study (Fuchs *et al*, 2014). Thus, clinical data predictive of response may vary between chemotherapy and targeted therapy and should be considered in both treatment and patient selection and be examined in more detail in trials of second-line therapy.

This meta-analysis has identified an OS benefit from second-line chemotherapy independent of age. Indeed, older age has been found to be a positive predictor of improved OS with second-line chemotherapy (HR=0.94 per 5 years, 95% CI=0.90–0.99). These findings, not previously reported in this setting, are of particular relevance for routine clinical practice considering the association of age with poor prognosis and increased cancer-specific mortality in patients with gastric cancers (Yang *et al*, 2011; Koppert *et al*, 2012). Furthermore, older age has, in some cases, been an exclusion criterion for trials in this setting (Thuss-Patience *et al*, 2011; Hironaka *et al*, 2013; Fuchs *et al*, 2014). The data from this meta-analysis suggest patients of older age have greater potential gain from second-line treatment and, consequently, their inclusion in future trials should be considered.

In a population vulnerable to both disease- and treatment-associated impacts on quality of life, future trials should include HRQoL outcomes to confirm improved OS is not achieved at the expense of reduced HRQoL. Of the phase 3 trials comparing second-line chemotherapy to SC, only Ford *et al* reported detailed HRQoL, and found no deterioration in global HRQoL and, furthermore, a reduction in pain with docetaxel and SC compared with SC alone (Ford *et al*, 2014). The REGARD and RAINBOW trials similarly found no adverse impact on global quality of life in treatment *vs* control arms. However, although at least 90% of patients completed baseline questionnaires in these studies, the proportion of patients completing questionnaires after 6 weeks of initiating treatment declined dramatically (Fuchs *et al*, 2014; Wilke *et al*, 2014). Overcoming the challenges of nonresponse will be important in the adequate assessment of quality of life.

The use of SC as a comparator arm in clinical trials requires effective definition and delivery to ensure validity. Across the three trials, patients in SC and SC plus chemotherapy arms were assessed with similar frequency, with attention given to both assessment and symptom management, conforming to consensus guidelines (Zafar *et al*, 2012). A potential limit to the general applicability of this meta-analysis is the median age of 59 years, younger than in the regular clinical population. However, this study is not limited by incomplete data retrieval and was informed by patient-level data of all three phase 3 trials. It therefore provides definitive guidance on the treatment of gastric and oesophageal adenocarcinoma with single-agent second-line chemotherapy. We note that similar HRs for OS with overlapping CI to this meta-analysis were published in a previous trial-level meta-analysis (Kim *et al*, 2013). The authors noted, however, that clinical and pathology data were not analysed and the outcome data for one of the trials were only reported in form of a conference abstract. Another meta-analysis, limited by the use of clinical trial-level data and inclusive of ramucirumab single agent treatment, reported similar results (Iacovelli *et al*, 2014). Although we largely agree with the discussion in both preliminary meta-analyses, we believe that a meta-analysis of phase 3 trials should be informed by the highest quality data at patient-level.

In conclusion, this meta-analysis demonstrates that second-line chemotherapy with docetaxel or irinotecan improves OS in gastric, gastroesophageal junction, and oesophageal cancers compared with SC alone. The wealth of evidence for efficacy of second-line treatment compared with SC, substantiated by this meta-analysis, confirms that second-line chemotherapy should be considered the standard of care. Future clinical trials in this setting should no longer consider SC an adequate control arm.

## Figures and Tables

**Table 1:** Study, patient, disease, and primary treatment characteristics

**Study characteristics**	**Ford *et al* (2014)** ***n*** **(%)**	**Thuss-Patience *et al* (2011)** ***n*** **(%)**	**Kang *et al* (2012)** ***n*** **(%)**	**Total**
Total patient enrolment	168	40	202	410
Treatment details	Docetaxel 75 mg m^−2^ every 3 weeks (*n*=84) *vs* SC (*n*=84)	Irinotecan 250 mg m^−2^ cycle 1, thereafter up to 350 mg m^−2^ every 3 weeks (*n*=21) *vs* SC (*n*=19)	Irinotecan 150 mg m^−2^ every 2 weeks (*n*=63) *vs* Docetaxel 65 mg m^−2^ every 2 weeks (*n*=70) *vs* SC (*n*=69)	
Maximum number of cycles	6	10	12	
**Patient characteristics**				
**Sex**				
Male	136 (81%)	29 (73%)	137 (68%)	302 (74%)
Female	32 (19%)	11 (22%)	65 (32%)	108 (26%)
**Age, years (median (range))**	65 (28–84)	58 (35–73)	56 (31–76)	59 (28–84)
Age ⩽70	129 (77%)	37 (93%)	182 (90%)	348 (85%)
Age >70	39 (23%)	3 (7%)	20 (10%)	62 (15%)
**ECOG PS**				
0 or 1	142 (84%)	31 (78%)	200 (99%)	373 (91%)
0	46 (27%)	–	108 (53%)	154
1	96 (57%)	–	92 (46%)	188
2	26 (16%)	9 (22%)	2 (1%)	37 (9%)
**Disease characteristics and previous treatment**				
**Disease status**				
Locally advanced	21 (13%)	0	0	21 (5%)
Metastatic disease	147 (87%)	40 (100%)	202 (100%)	389 (95%)
**Site of primary disease**				
Oesophagus	33 (20%)	0	0	33 (8%)
Oesophago-gastric junction	59 (35%)	17 (42%)	0	76 (18%)
Stomach	76 (45%)	23 (58%)	202 (100%)	301 (73%)
**Response to previous CT**				
CR	0	2 (5%)	4 (2%)	6 (1%)
PR	29 (17%)	9 (22%)	79 (39%)	117 (29%)
SD	42 (25%)	10 (25%)	26 (13%)	78 (19%)
PD	91 (54%)	19 (48%)	89 (44%)	199 (49%)
Non-evaluable	6 (4%)	0	4 (2%)	10 (2%)
**Time between end of previous CT and documented PD**				
During treatment	72 (43%)	16 (40%)	74 (37%)	162 (39%)
Within 3 months	49 (29%)	19 (48%)	75 (37%)	143 (35%)
3–6 Months	47 (28%)	5 (12%)	53 (26%)	105 (26%)
**Number of sites of PD**				
1	65 (39%)	17 (42%)	70 (35%)	152 (37%)
2 or more	103 (61%)	23 (58%)	132 (65%)	258 (63%)
**Previous surgery**				
No	127 (76%)	20 (50%)	158 (78%)	305 (74%)
Yes	41 (24%)	20 (50%)	44 (22%)	105 (26%)
**Previous radiotherapy**				
No	157 (93%)	40 (100%)	172 (85%)	369 (90%)
Yes	11 (7%)	0	30 (15%)	41 (10%)

Abbreviations: CR=complete response; PD=progressive disease; PR=partial response; PS=performance status; SD=stable disease.

**Table 2:** Chemotherapy and disease response characteristics

			**Kang *et al* (2012)**		
**Chemotherapy details**	**Ford *et al* (2014)** ***n*** **(%)**	**Thuss-Patience *et al* (2011)** ***n*** **(%)**	**Total,** ***n*** **(%)**	**Docetaxel arm,** ***n*** **(%)**	**Irinotecan arm,** ***n*** **(%)**
CT+SC	84	21	133	70	63
**CT type**					
Docetaxel	84 (100%)	0	70 (51%)		
Irinotecan	0	21 (100%)	63 (47%)		
**Number of cycles administered**					
0	7 (8%)	2 (10%)	2 (2%)	1 (1%)	1 (2%)
1	17 (20%)	2 (10%)	20 (15%)	11 (16%)	9 (14%)
2	10 (12%)	7 (33%)	29 (22%)	20 (29%)	9 (14%)
3	23 (27%)	2 (10%)	23 (17%)	13 (19%)	10 (16%)
4	5 (6%)	2 (10%)	10 (8%)	4 (6%)	6 (10%)
5	3 (4%)	3 (14%)	6 (5%)	1 (1%)	5 (8%)
6	19 (23%)	1 (5%)	22 (17%)	13 (19%)	9 (14%)
>6	0	2 (10%)	21 (16%)	7 (10%)	14 (22%)
Total number of cycles	255	68	543	243	300
Median number of cycles (range)	3 (0–6)	2 (0–9)	3 (0–12)	3 (0–9)	4 (0–12)
**Reason for treatment ending**					
Completion of treatment	19 (23%)	0	0	0	0
Unacceptable toxicity	20 (24%)	2 (10%)	2 (1%)	2 (3%)	0
Treatment delay >21 days	5 (6%)	0	1 (1%)	1 (1%)	0
Progressive disease	26 (31%)	13 (62%)	126 (95%)	67 (96%)	59 (94%)
Treatment refused by patient	1 (1%)	1 (5%)	4 (3%)	0	4 (6%)
Patient died	10 (12%)	2 (10%)	0	0	0
Other	3 (4%)	3 (14%)	0	0	0
**Best response to CT**					
Partial response	4 (5%)	0	11 (8%)	6 (9%)	5 (8%)
Stable disease	26 (31%)	10 (48%)	39 (29%)	18 (26%)	21 (33%)
Progressive disease	24 (29%)	11 (52%)	81 (61%)	46 (66%)	35 (56%)
Non-evaluable/not assessed	30 (36%)	0	2 (2%)	0	2 (3%)

Abbreviations: CT=chemotherapy; SC=supportive care.

**Table 3:** Summary of OS and hazard ratios

**Survival**	**Ford *et al* (2014)** ***n*** **(%)**	**Thuss-Patience *et al* (2011)** ***n*** **(%)**	**Kang *et al* (2012)** ***n*** **(%)**
Total patient enrolment	168	40	202
Number of events	165	40	202
**Median survival (95% CI)**			
CT+SC	5.2 (4.1–5.9)	4.0 (2.6–5.6)	6.3 (5.0–7.2)
SC	3.6 (3.3–4.4)	2.4 (1.2–3.5)	3.7 (2.7–4.5)
Hazard ratio (95% CI)	0.72 (0.53–0.98)	0.48 (0.25–0.92)	0.58 (0.43–0.78)
*P*-value (two sided)	0.04	0.02	0.0003

Abbreviations: CI=confidence interval; CT=chemotherapy; OS=overall survival; SC=supportive care.

**Table 4:** Treatment by covariate interactions in a one-stage random effects Cox regression model for OS

**Parameter**	***P*****-value for treatment by covariate interaction term**
Age	0.34
Gender (male *vs* female)	0.88
Performance status (0 or 1 *vs* 2)	0.44
Disease stage (locally advanced *vs* metastatic)	0.18
TTP from first-line chemotherapy (during treatment *vs* within 3 months *vs* within 3–6 months)	0.04
Disease site (oesophagus *vs* OG junction *vs* stomach)	0.28
Number of progression sites (1 *vs* 2 or more)	0.28

Abbreviation: TTP=time to progression.
