# Supplementary material for: Chemotherapy vs supportive care alone for relapsed gastric, gastroesophageal junction, and oesophageal adenocarcinoma: a meta-analysis of patient-level data
Source: Br J Cancer. 2016 Feb 16;114(4):381–7. doi: 10.1038/bjc.2015.452 (PMC4815769; doi:10.1038/bjc.2015.452)
Supplement: Supplementary Table S1 [file bjc2015452x2.docx]

Supplementary Table 1: Patient monitoring

|  | **Monitoring within both arms** | **Palliative Radiotherapy allowed?** |
| --- | --- | --- |
| **Thuss-Patience *et al*, 2011** | Weekly assessment, including full blood count (FBC)/ biochemistry. After treatment termination, assessment every three weeks | Yes |
|  |  |  |
| **Kang *et al*, 2012** | Reviewed every two to three weeks | Yes |
|  |  |  |
| **Ford *et al*, 2014** | Assessment every three weeks (or prior to each cycle) during treatment period, including FBC/ biochemistry. After treatment period, assessment every 6 weeks for up to one year and then assessment every three months. | Yes |
|  |  |  |
