# Supplementary material for: Chemotherapy vs supportive care alone for relapsed gastric, gastroesophageal junction, and oesophageal adenocarcinoma: a meta-analysis of patient-level data
Source: Br J Cancer. 2016 Feb 16;114(4):381–7. doi: 10.1038/bjc.2015.452 (PMC4815769; doi:10.1038/bjc.2015.452)
Supplement: Supplementary Table S2 [file bjc2015452x3.docx]

**Supplementary Table 2** Number of patients receiving additional CT

| **Study Characteristics** | **Ford *et al.***  **(2014)** | | **Thuss-Patience *et al.* (2011)** | | **Kang *et al.***  **(2012)** | |
| --- | --- | --- | --- | --- | --- | --- |
|  | **n (%)** | | **n (%)** | | **n (%)** | |
|  | CT+SC | SC | CT+SC | SC | CT+SC | SC |
| **Number of patients** | 84 | 84 | 21 | 19 | 133 | 69 |
| **Further chemotherapy** |  |  |  |  |  |  |
| **No** | 77 (92%) | 69 (82%) | 18 (86%) | 17 (89%) | 78 (59%) | 54 (78%) |
| **Yes** | 7 (8%) | 15 (18%) | 3 (14%) | 2 (11%) | 55 (41%) | 15 (22%) |
