# Supplementary material for: Chemotherapy vs supportive care alone for relapsed gastric, gastroesophageal junction, and oesophageal adenocarcinoma: a meta-analysis of patient-level data
Source: Br J Cancer. 2016 Feb 16;114(4):381–7. doi: 10.1038/bjc.2015.452 (PMC4815769; doi:10.1038/bjc.2015.452)
Supplement: Supplementary Materials [file bjc2015452x4.docx]

Supplementary Material

**Supplementary Table 1** Patient monitoring

**Supplementary Table 2** Number of patients receiving additional CT

**Supplementary Figure 1** Kaplan-Meier survival curve. This is for illustration and represents the pooled data from the three trials. No Hazard Ratios have been generated from these data. Instead, we refer the reader to the definitive hazard ratios in the text.(Tierney *et al*, 2015).
